# Health care workers’ experiences of managing foetal distress and birth asphyxia at health facilities in Northern Uganda

**DOI:** 10.1186/s12978-021-01083-1

**Published:** 2021-02-05

**Authors:** Elizabeth Ayebare, Grace Ndeezi, Anna Hjelmstedt, Jolly Nankunda, James K. Tumwine, Claudia Hanson, Wibke Jonas

**Affiliations:** 1 Department of Nursing, School of Health Sciences, College of Health Sciences, Makerere University, Kampala, Uganda; 2 Department of Paediatrics and Child Health, School of Medicine, College of Health Sciences, Makerere University, Kampala, Uganda; 3 Department of Women’s and Children’s Health, Karolinska Institutet, Stockholm, Sweden; 4 Mulago Specialized Women’s and Neonatal Hospital, Kampala, Uganda; 5 Department of Public Health Sciences, Karolinska Institutet, Stockholm, Sweden; 6 Department of Disease Control, London School of Hygiene and Tropical Medicine, London, UK

**Keywords:** Birth asphyxia, Foetal distress, Healthcare workers, Low income setting, Midwives, Referral systems

## Abstract

**Background:**

Birth asphyxia is one of the leading causes of intrapartum stillbirth and neonatal mortality worldwide. We sought to explore the experiences of health care workers in managing foetal distress and birth asphyxia to gain an understanding of the challenges in a low-income setting.

**Methods:**

We conducted in-depth interviews with 12 midwives and 4 doctors working in maternity units from different health facilities in Northern Uganda in 2018. We used a semi-structured interview guide which included questions related to; health care workers’ experiences of maternity care, care for foetal distress and birth asphyxia, views on possible preventive actions and perspectives of the community. Audio recorded interviews were transcribed verbatim and analysed using inductive content analysis.

**Results:**

Four categories emerged: (i) Understanding of and actions for foetal distress and birth asphyxia including knowledge, misconception and interventions; (ii) Challenges of managing foetal distress and birth asphyxia such as complexities of the referral system, refusal of referral, lack of equipment, and human resource problems, (iii) Expectations and blame from the community, and finally (iv) Health care worker’ insights into prevention of foetal distress and birth asphyxia.

**Conclusion:**

Health care workers described management of foetal distress and birth asphyxia as complex and challenging. Thus, guidelines to manage foetal distress and birth asphyxia that are specifically tailored to the different levels of health facilities to ensure high quality of care and reduction of need for referral are called for. Innovative ways to operationalise transportation for referral and community dialogues could lead to improved birth experiences and outcomes.

## Background

Globally, Birth Asphyxia (BA) accounts for 23 percent of all neonatal deaths [1, 2]. Birth asphyxia is defined as failure of the newborn to initiate and sustain breathing at birth or an Apgar score of less than 7 at 1 min [3, 4]. Birth asphyxia occurs due to interruption of placental blood flow leading to foetal hypoxia and acidosis. This may manifest with abnormalities in the foetal heart rate also known as foetal distress [5]. In the first days of life, BA may lead to generalized organ damage such as acute kidney injury while long term complications may include infant neurological disorders or cognitive impairment [6, 7].

In Uganda, BA accounts for 28 percent of the neonatal deaths currently at 27/1000 live births [8]. The major factors contributing to BA related mortality have been shown to include maternal delay to seek care, staff’s poor interpretation of signs of foetal distress during labour, inadequate foetal monitoring and inappropriate response to poor progress in established labour [9]. The *Every Newborn Lancet Series* points to the relevance of good quality of intrapartum care as a key contributor to reduce BA and neonatal mortality with interventions such as skilled attendance at birth, promotion of birth preparedness, basic and comprehensive obstetric care and essential newborn care including neonatal resuscitation [10, 11]. Skilled attendance at birth is therefore a precursor that is needed before implementing other interventions to reduce neonatal mortality. In a global review, it was estimated that improving the quality of facility based care during labour would avert 73 percent of all newborn deaths by 2025 [2]. In Uganda, skilled attendance at birth increased to 74 percent in 2016 [8], which creates an opportunity to reduce newborn deaths by providing quality intrapartum care.

In Northern Uganda, the recent conflict and presence of humanitarian emergencies compounded with weak health systems, have increased the risk of newborn deaths [12]. The Northern Uganda region is still recovering from nearly 20 years of conflict due to the Lord’s Resistance Army rebel group that ended in 2006 [13]. During the conflict (2001–2006), the neonatal mortality rate in the region was the highest in the country at 42/1000 live births [14]. Although the mortality rate had decreased to 32/1000 live births in 2016, this region still has one of the highest neonatal mortality rates in the country [15]. Few studies have looked at the management of foetal distress and BA in health facilities in this region. This study therefore aimed at exploring health care workers’ experiences of managing foetal distress and birth asphyxia to gain an understanding of the challenges and gaps in care and to make recommendations for interventions to reduce the rates of birth asphyxia related morbidity and mortality.

## Methods

### Study design

This was a descriptive qualitative study using in depth interviews to explore health care workers’ experiences of managing foetal distress and birth asphyxia. According to Bradshaw, Atkinson and Doody, qualitative descriptive research seeks to provide rich descriptions of a phenomenon on which little is known. We aimed to gain an understanding of management of foetal distress and BA from the health care worker’s experiences, understanding and viewpoints. Data was collected from participants’ natural places of work and in the initial analysis, the researchers sought to gain insight into the experiences of participants as described and expressed through the transcripts [16].

### Study setting and guidelines

The study was conducted in five public health facilities in Northern Uganda from February to June 2018. Below are the characteristics of the health facilities included:

1. A referral hospital which had ten midwives, one consultant obstetrician/gynaecologist and two medical officers allocated to the maternity unit at the time of the study. Comprehensive obstetric and newborn care is available including caesarean section, vacuum extraction, resuscitation and neonatal special care unit facilities. The hospital’s maternity unit has approximately 4500 births annually.
2. One Health Centre IV was included with a maternity section served by two doctors, three midwives, an anaesthetist and two laboratory technicians. The doctors and laboratory technicians also serve the rest of the health facility departments. This is the first level of referral for maternal and newborn health problems in Uganda and has the ability to perform caesarean sections. It has an ambulance which is shared by all the attached Health Centre III facilities when referral is needed. Ideally, this level of facility has blood transfusion services but at the time of data collection, this was not available. Annually, around 1000 infants are born at the Health Centre. The facility has a postnatal ward where mothers and newborns are observed after birth until discharge. There is no special care unit for sick newborns.
3. Three Health Centre III facilities were selected by simple random sampling from a total of five facilities. This level of facility has admission services including a labour ward and an admission ward where care is provided for antenatal, early labour and postnatal mothers. The facilities are headed by a clinical officer, has one nursing officer and one or two midwives to provide maternity care. At these facilities, the average number of births is approximately 250 annually.

### Uganda Clinical Guidelines (UCG) for management of foetal distress

There are no specified guidelines on management of foetal distress however during labour; health care workers should monitor labour using the partograph and consider a foetal heart rate of less than 120 bpm and higher than 160 bpm as foetal distress. When the woman is undergoing induction of labour and gets hypertonic contractions or foetal distress; the UCG requires the health care workers to (1) stop the oxytocin infusion, (2) give Salbutamol 5 mg in 500 ml of Normal saline at a rate of 10 drops per minute, and/or (3) perform an emergency caesarean section. Foetal distress may also occur when labour is obstructed. In this case, the health care workers should (1) give intravenous (IV) fluids to the mother to prevent dehydration and ketosis, (2) antibiotics for prophylaxis and (3) refer to a higher level facility [17].

### Uganda Clinical Guidelines (UCG) for management of BA

In Uganda,  BA is defined  by Apgar scores of less than or equal to seven at one minute of birth as stipulated in the WHO guidelines. The UCG on resuscitation to manage BA state that the newborn should be dried and stimulated.  In case there is no response,  the cord is clamped and cut to enable the health care workers transfer the newborn to a safe place for resuscitation. The mother should be informed about the condition of her newborn. The health care workers should ensure that the airway of the newborn is free and perform suction of mouth and nose to remove secretions. The guidelines caution health care workers not to suction too deep to avoid further problems. The newborn shall then be ventilated until the breaths are more than 30 per minute. If this does not happen, ventilations should be continued at a rate of 40 per minute. If available, oxygen should be provided. The newborn may be pronounced dead after 20 min of resuscitation without improvement. The guidelines discourage chest compressions citing that it could cause problems if not performed properly. Use of drugs is not considered necessary [17].

### Study population, sample size and data collection procedures

Sixteen midwives and doctors were purposively selected from the health professionals working in the maternity units at the health facilities. We considered characteristics such as years of experience in maternity care, level of education, type of professional and level of health facility in order to achieve maximum variation. The health care workers were approached at the health facilities and informed about the study. Those who expressed interest in taking part in the study were asked for an appointment date for the interview. A semi-structured interview guide was developed from a review of literature by the authors. The interview guide included questions about education and work experience of the health care workers, their experience of management of foetal distress and BA, facilitators and barriers to management of the above conditions, how BA could be prevented and community attitudes regarding management of foetal distress and BA. The interviews were conducted by EA, a midwife and one trained BSc. Nurse, in a quiet room selected by the health care workers at the facility and lasted for approximately 30–45 min. All interviews were conducted in English and audio recorded to ensure that all information was captured. For establishing a sufficient sample size, we considered the aim of the study, the nature of the phenomenon under investigation and the quality and richness of the data obtained [18, 19]. We achieved data saturation after 16 participants had been interviewed as no new information was coming out of the interviews.

### Data management and analysis

Interviews were transcribed verbatim and the transcripts stored on a password protected computer only accessible to the principal investigator and members of the research team. Data were analysed by inductive content analysis suggested by Elo and Kyngäs [20] where categories are identified from the data, analysed and reported. To begin with, full interview transcripts were read and re-read by EA and AH to get a sense of the whole data. Then, each transcript was used as a unit of analysis to perform open coding together with categorization and abstraction of quotes. The codes were then grouped into wider sub-categories and initial categories were determined by EA, AH and WJ. Final categories were reviewed and agreed upon by AH, WJ, CH and GN as presented in the results. The results were continuously discussed within the research group until consensus regarding the interpretations was reached. None of the authors has worked in the specific health facilities where the study was conducted although EA has worked in a similar setting in Uganda. The group of authors consists of midwives, an obstetrician, paediatricians and a neonatologist. This diversity among the authors allowed for deeper discussions and richer perspectives in the final results.

### Ethical considerations

Ethical approval was obtained from Makerere University School of Health Sciences Research and Ethics  Committee (SHSREC 2017-051) and the Uganda National Council for Science and Technology. Administrative clearance was obtained from the District Health Office and the participating health facilities. All participants gave written informed consent and permission to audio record the interviews before participating in the study.

## Results

### Demographic characteristics of health care workers

Participants were 12 midwives and four doctors including one specialized in obstetrics and gynaecology. Participants’ ages ranged from 23 to 48 years. All participants were trained in newborn resuscitation and 12 of them had received the training within less than six months from the interview. Thirteen of the health care workers had worked in a maternity unit for a period of more than a year. Details of the participants’ socio-demographic characteristics are shown in Table 1.

**Table 1:** Demographic characteristics of participants

Characteristics	Frequency
*Age category*	
20–29	8
30–39	5
40–49	3
*Sex*	
Male	4
Female	12
*Health care workers’ qualification*	
Diploma midwife/nurse	6
Certificate midwife	6
General doctor	3
Specialist doctor (obstetrician/gynaecologist)	1
*Health care workers’ level of health facility*	
Hospital	10
Health centre IV	1
Health centre III	5
*Years of experience in maternity care*	
Less than 1 year	3
1–5 years	8
6–10 years	3
More than 10 years	2
*Time since training in neonatal resuscitation*	
Six months or less	12
7 months to 1 year	2
More than 1 year	2

### Categories

In the following section, we present the main categories that emerged from the data and illustrate these with quotes from our interviewees. Data analysis yielded the following four categories: (i) Understanding of and actions for foetal distress and BA, (ii) Challenges of managing foetal distress and BA, (iii) Expectations and blame from the community, and finally (iv) Health care workers’ insights into the prevention of BA. A diagrammatic presentation of the analysis for one category is presented as Table 2 in Appendix 1.

### Category 1: understanding of and actions for foetal distress and birth asphyxia

#### Knowledge regarding foetal distress

Health care workers defined foetal distress according to the Ugandan guidelines. In some cases, different cut off values for the foetal heart rate were given such as above 150 or below 110 beats per minute. To confirm the signs of foetal distress, health care workers monitored the foetal heart between and immediately after the contractions, and more frequently to ensure that the diagnosis was right.

“A normal foetal heart ranges from 120 to 160. So when the foetal heart is below or above that range then it means the foetus is going to distress. That’s why we always have to listen to the foetal heart the moment they come. And that’s why it’s important for us to count to know the normal from the abnormal one.” Midwife#14, Health Centre III.

#### Interventions for foetal distress

Rehydrating mothers was the most common action that was taken to manage foetal distress by giving intravenous (IV) fluids such as Normal Saline and 5% Dextrose. Participants reported giving fluids as the first intervention because most mothers were thought to come to the facility dehydrated or very hungry. However, it was noted that some health care workers would delay to refer women for up to two hours while waiting for the foetal heart rate to normalise during intravenous fluid administration. Lateral positioning and giving oxygen were mentioned as other ways to manage foetal distress. Women were referred to the health centre IV or the hospital for a caesarean section when the initial interventions such as giving fluids and change of position failed to lead to normalisation of the foetal heart rate during the first stage of labour. During the second stage of labour, health care workers reported that they administer IV fluids, augment the labour with oxytocin and encourage the mother to push to enable quick delivery. Rarely, births were completed using vacuum extraction.

“…if you identify a mother who is having foetal distress you monitor more frequently like the foetal heart rate with your Pinard [Fetoscope] at the same time you start some other intervention and put on an IV fluid put the mother on the lateral side and then you start monitoring the foetal heart rate sometimes they improve depending on the cause.” Doctor#2, Hospital.

#### Knowledge about birth asphyxia

Health care workers described BA as low Apgar scores of less than 7 or when the infant is born and does not cry. The timing when the Apgar score would be considered BA was rarely specified. Statements like *“when the baby is not breathing well”*, or even *“failure to gain normal respiration”* or *“failure to initiate breathing”* were used to define BA. Health care workers mentioned signs and symptoms such as chest in-drawing, nasal flaring, grunting, gasping, blue skin colour and lack of cord pulsations as indicators of BA. There was an attempt to classify the degree of severity of BA using the Apgar score. Generally, babies with Apgar scores of 3 or less were said to be difficult to resuscitate due to severity of BA.

“Birth asphyxia is when a baby is born then it is having a score of less than 7. A baby may come out and doesn’t cry immediately after birth, the baby is blue, the muscle tone is poor, doesn’t feel anything –if you touch it doesn’t feel-the senses are not there, definitely that baby will have asphyxia.” Midwife#5, Health Centre III.

#### Interventions for birth asphyxia

Health care workers performed resuscitation using an ambu bag and bulb syringe to manage BA. Stimulating the newborn, suctioning, and positive pressure ventilation using the ambu bag were mentioned as aspects of resuscitation. In newborns with meconium stained liquor, suctioning was said to be the first option while other health care workers prioritized providing warmth. There seemed to be a lack of clarity on how deep to suction during resuscitation.

The urgency of conducting the resuscitation to prevent severe asphyxia was expressed through the narratives of the health care workers.

“…the first thing you have to provide warmth to this baby, then you check the airway if it is meconium you suck it away. You do suction and normally during suction you have a stimulator to see if it can pick up, if after all this you fail then you start bagging [bag and mask ventilation] the baby. Another thing is to act as quickly as possible.” Midwife#13, Hospital.

Giving 10% dextrose to newborns was said to help babies who are not breathing since they could be having hypoglycaemia. Using drugs including aminophylline, dexamethasone and atropine during resuscitation was frequently mentioned by midwives at the Health Centre III level. Oxygen was also considered helpful during resuscitation.

“Maybe if you do that and it fails maybe; it’s hypoglycaemia that is making the baby like that because with hypoglycaemia there will be reduced oxygen supply to the brains, so the moment you give dextrose it might also improve”. Midwife #12, Health Centre III.

### Category 2: challenges of managing foetal distress and BA

#### Complexities of the referral system

One of the major challenges to managing both foetal distress and BA was the referral system. Health care workers called upon the sub-county ambulance based at the Health Centre IV to be available to transfer women. However, the one ambulance was not sufficient to serve all the health centres in the catchment area. In addition, lack of fuel, ambulance breakdown, and lack of drivers were commonly reported which lead to referral of women and newborns using motorcycles (boda-bodas). In other facilities, midwives waited for private or public transport vehicles on the road side to take the referred mothers to the next level of care and paid for transportation of the woman.

Health care workers did not have the possibility to escort the mothers when they worked alone at the facility or when the mother had to go by a motorcycle so they followed-up by phone. Due to the limited resources at the first level and other levels of referral, health care workers preferred to refer to the regional referral hospital directly.

“Sometimes you call the ambulance and they have no fuel so these mothers have to go on bodas (motorcycles)… You know it’s not easy in the darkness [at night]; those are challenges.” Midwife #15, Health Centre IV.

#### Refusal of referral

Another challenge was that mothers with foetal distress themselves sometimes declined referral to higher level facilities. Therefore, health care workers had to convince and explain to the women that the lower health centre did not have the ability to manage foetal distress. Some of the reasons why mothers refused referral were; lack of money to cater for the personal costs of being admitted and having had an established relationship with their midwife at the lower level facility which lead to reluctance in being cared for by a different health care worker.

“First of all, mothers themselves are really an issue. Sometimes they refuse the referrals-they don’t want to go. So it makes us delay with the mother –convincing, delaying, talking… You find that a mother whom you have talked to about referring during antenatal; labour starts and she comes here, …like there was one who had previous uterine scar, she came here yet we had referred her. She told us [that] she wants us to first try…”. Midwife#14, Health Centre III.

#### Lack of supplies and equipment

Limited resources made it difficult to offer appropriate care for foetal distress and BA. At the lower level facilities, participants mentioned lack of oxygen as a challenge during and after newborn resuscitation. This meant that midwives have to refer unstable newborns to a facility 20–25 kms away to receive oxygen. At the Health Centre IV level, an oxygen concentrator was used to provide oxygen to newborns. This oxygen concentrator uses a solar power source, which is unreliable because its power gets depleted often especially at night. The inadequate power supply not only affected oxygen delivery but also lighting to perform other procedures at night such as emergency caesarean sections or inserting intravenous lines.

“I think, more so like in our case, we may have a gap in management of asphyxia like we don’t have oxygen, so for the babies we refer, if at all we had oxygen we could keep them here.” Midwife #5, Health Centre III.

“Our theatre is there, fully functioning but there is no equipment. […] Here we have no blood bank and its risky. You can’t have an operation without a blood bank…” Midwife #15, Health Centre IV.

Equipment for resuscitation were available but not always in proper condition for immediate use. Health care workers pointed out situations when all equipment were unsterile yet there was a newborn with BA. They perceived the lack of drugs such as aminophylline, dexamethasone and 10% dextrose as a barrier to effective care for babies with BA. A special room for neonatal care was said to be lacking in lower level facilities including the Health Centre IV. Because of this, sick babies were managed in the labour ward or postnatal ward with many other mothers around and no considerations for warmth which put the small for age and preterm babies at an increased risk of death. Continuity of care after resuscitation was inadequate and so some babies deteriorate even after successful resuscitation.

“There is need for a special place for babies with birth asphyxia during observation period. This is not available at most health centre IIIs.” Midwife #14, Health Centre III.

“…because babies normally die after resuscitation you resuscitate a baby and comes up very well now the continuous care tend[s] to forget [be forgotten] …” Doctor#2, Hospital.

#### Human resources challenges

Health care workers talked about staff shortage and absence from duty as a barrier to appropriate care. Sometimes, they had to call upon other non-competent staff members to assist during neonatal resuscitation. For example, a midwife called upon a nurse or nursing assistant to help with newborn resuscitation while she managed postpartum haemorrhage. In cases where both the newborn and mother experienced complications the health care workers were in a dilemma to decide who would get attention first. Working alone was a recurring challenge at all levels of care. Team work was reported to be important, yet in many situations, participants said it was difficult to achieve due to understaffing or absence of responsible health care workers from the facility. Consultations were made by phone calls to seniors or colleagues sometimes using the health care worker’s personal phone and airtime. Health care workers sometimes had to independently make decisions to ensure quick interventions for mothers with emergencies.

“The number of staff; like if you are on duty and you are only one, so handling two people at ago because when the baby comes out with asphyxia the baby needs you and the mother still here also needs you so that is the main challenge.” Midwife#6, Health Centre III.

### Category 3: expectations and blame from the community

Participants reported that the community’s expectation is for every pregnant woman to leave the hospital with a live infant despite the condition of the mother. Health care workers therefore felt under pressure from the community and caretakers to achieve this expectation. Ensuring that babies were born alive was described as hard work and a struggle by health care workers to save lives. Sometimes, health care workers were blamed by attendants and family members for causing the BA. To give an example, if the midwife was rude or not available at the facility or did not act quickly, she would be blamed. Therefore, health care workers tried to have an effective and continuous communication with the attendants and the mother in order to create trust and reduce incidences of blame.

“They expect us to work so hard and deliver a live baby, they expect that we have to do something, and that if the baby comes out like that [with birth asphyxia] we have what it takes to save the baby.” Midwife#14, Health Centre III.

“And sometimes when you leave the attendants out and you are alone with the mother and then the mother delivers and the baby has asphyxia they will blame you the midwife.” Midwife #5, Health Centre III.

Participants reported that communities perceived BA to be caused by witchcraft or due to something wrong that the mother did during pregnancy. Therefore, the blame was apportioned to the mothers. The caretakers believe that if a mother does not push adequately, she is lazy or she was inactive during pregnancy; this could cause her infant to be born with asphyxia. Caretakers (mostly mothers’ in law) were said to abuse the mothers by slapping or threatening them that the infant will die if they do not push. Midwives reported that relatives can even ask for an episiotomy to be performed if they think the mother is not pushing adequately.

“If the mother was not pushing enough they will blame the mother directly and sometimes they even end up slapping the mother in the labour ward. ‘If you don’t push the baby, you end up going home with only clothes and the like and they don’t want that to happen so they encourage the mother to push harder… Sometimes they tell me[midwife] that if the mother is not pushing you cut her [do an episiotomy] …” Midwife#5, Health Centre III.

### Category 4: health care workers’ insights into prevention of foetal distress and birth asphyxia

To prevent BA, health care workers emphasized that mothers should be taught to come early in labour and not in second stage. Ensuring that mothers understand the signs of labour and when to seek health care was also mentioned to be important. Further, the study participants said that it is key to screen women from early on and to identify and manage risk factors for BA such as pre-eclampsia, malaria and anaemia. Health care workers strongly emphasised that adolescent pregnancies should be avoided since BA was more common among younger mothers.

“… only that we need to sensitize the community for early antenatal, strengthen school programs to adolescents to avoid early pregnancies…” Midwife#15, Health Centre IV.

“Screening mothers with risk factors from antenatal and then when you get them, you have to explain to them the impact of child birth and then you make early referral to a higher level of management. Then we can also treat some conditions early; like pre-eclampsia…” Midwife#16, Health Centre III.

Preventive measures suggested during labour included proper hydration of mothers by encouraging them to drink tea and other fluids. Proper labour monitoring, pelvic assessment and taking action in case of any abnormality was another way to prevent asphyxia. Companionship during labour was said to reduce stress and BA. Health care workers at the referral facility recounted that they had not been preventing BA but only fighting it after it has happened. This was due to the late referrals with already existing complications. At this level, skills in newborn resuscitation were said to be the only way to prevent complications of BA.

## Discussion

### Summary of findings

In the present study, we sought to identify health care workers’ experiences of managing foetal distress and BA at different levels of health care in Northern Uganda through qualitative interviews.

Health care workers find themselves in a difficult situation with inadequate resources, and internal pressures that affect their ability to offer good quality care. The lack of resources included; oxygen for use during neonatal resuscitation at Health centre III, a special care area for newborns with BA at Health centre III and IV and blood for transfusion during caesarean section at Health centre IV and the hospital. The referral process was equally affected by the inadequate resources at each level of care and problems with transportation. We found that, although most of the study participants are able to assess foetal distress and BA, there were some misconceptions in the management such as delayed referral of mothers and use of drugs during newborn resuscitation which may lead to poor outcomes. Health care workers also felt under pressure to perform beyond their abilities due to high expectations from women and their surrounding community. Our study also shows that women in labour were blamed and abused by relatives when they gave birth to a newborn with BA.

### Health system challenges

At the lower level facilities, we identified challenges such as lack of oxygen, and lack of special care units for sick newborns that affect the management of BA. This is in line with another study conducted in Northern Uganda (2015) that showed that the Health Centre IV as the first level of choice for referral was found to be unprepared to provide care for women with foetal distress and newborns with BA due to unreliable supply of oxygen, absenteeism of health care workers and lack of blood [21]. Due to this lack of preparedness, midwives at lower levels referred mothers directly to the referral hospital and by doing so, bypassing the existing referral pathways.

The Uganda health system assessment report 2011 showed similar patterns of referral attributed to lack of adequate resources to handle possible complications [22].

Our data suggest that even at the lowest health facility where intrapartum care is provided, a special care unit could ensure that sick newborns are provided with essential care such as warmth, oxygen, and support for breastfeeding. In addition, more affordable and effective ways to provide warmth such as skin to skin care and breastfeeding could be emphasized at lower level facilities to reduce the need for referrals and deaths among newborns with BA [23, 24]. It is therefore important, to reassess the essential services needed at each level of care to ensure proper management of mothers with foetal distress and newborns with BA. At the facilities in the present study setting, there was poor continued care after newborn resuscitation yet, this is vital to ensure survival. Literature shows that most newborns with low Apgar scores at birth need observation for at least 24 h before discharge [25] to avoid further complications and newborn death.

With respect to the challenge with transport and referral when foetal distress and BA occurred, our study adds to the plethora of literature describing the challenges of emergency referral in LMIC [26]. Available services at health facilities seem not to be adequately utilised because of external factors such as a poor transport system, inadequate supplies and equipment as well as human resource challenges [21, 27]. While these challenges still exist in many low income countries [28, 29], referral of women with complications is an essential facilitator for quality maternal and newborn care that must be given attention. Several studies have shown that obstetric referrals could be facilitated by prepaid vouchers to improve birth outcomes [30]. This initiative could be taken up by the Ministry of Health to test its ability to improve birth outcomes. In this study, referral processes were further complicated by the mother’s refusal to comply with the decision to go to the next level of care. Studies have attributed this to cultural beliefs, poverty and poor decision making ability of the woman in labour [21, 26].

### Interventions for foetal distress and birth asphyxia

In general, staff knowledge of management of foetal distress and BA was good but there were some misconceptions. We noticed lack of clear guidance on effective management of foetal distress which might be partly attributed to the lack of specific guidelines in Uganda. To give a few examples, health care workers reported to administer intravenous fluids to the mother when foetal distress was detected. The role of intravenous fluids according to the UCG is for rehydration and prevention of ketosis for women in labour irrespective of their foetal heart rate status [17] and similarly, intravenous fluids are provided as the first action for foetal distress in high and other low income countries [31–34]. However, WHO guidelines do not support administration of intravenous fluids as an action against foetal distress [35]. Our study shows that administration of intravenous fluids may have resulted in delays of up to two hours in making the decision to refer women with foetal distress which  could lead to poor outcomes.

Lateral positioning of the mother to manage foetal distress reported in this study has been widely documented in other studies and is also recommended by WHO guidelines [31, 32, 35, 36]. Although oxygen administration to the mother is reported as an intrauterine resuscitation measure, it is not recommended for use neither in the Ugandan nor in the WHO guidelines [17, 35]. In addition, the benefit of using oxygen for intrauterine resuscitation when there is foetal distress has not been clearly established [37, 38]. Since management of foetal distress should target the potential cause, several context specific interventions should be recommended.

Health care workers managed BA by following guidelines for neonatal resuscitation as recommended in the UCG [17]. The UCG guidelines and the helping babies breathe (HBB) algorithm for neonatal resuscitation recommend a focus on positive pressure ventilation since a majority of newborns with BA will improve without chest compressions [39]. A study in Kenya showed that 86% of all newborns requiring resuscitation survived after performing drying, stimulations, suctioning and positive pressure ventilation [40]. Surprisingly, we found that, health care workers commonly mentioned the need for drugs such as aminophylline, adrenaline and atropine for use during neonatal resuscitation. These drugs could lead to deterioration of the newborn when used and are not recommended especially for preterm newborns [41]. Administration of adrenaline also requires that a specialist neonatologist or paediatrician is available and that facilities to offer advance life support are present. In addition, only one percent of newborns with BA may require use of drugs during resuscitation [42]. This implies that use of drugs for neonatal resuscitation may not be beneficial at lower level facilities.

### Blame and mistreatment

Our results also show that relatives blamed mothers and sometimes health care workers when BA happened. This put health care workers under significant pressure. Health care workers have been blamed by family members when poor newborn outcomes such as stillbirths occur attributing it to disrespectful and delayed care [43]. However, the blame may sometimes be wrongly apportioned due to the family’s lack of information about the cause of BA or any other poor outcome. Health care workers reported that companions, typically the mother-in-law, shouted or slapped the parturient to ensure improved pushing as shown in a previous study in this setting [21]. While several studies have been reporting such mistreatment and abuse among health care providers, abuse by the companion has not been described [45]. This is a tragedy, since abuse and disrespectful care leads to poor birth outcomes and impacts the mother’s psychological wellbeing [46]. It also means that decision making ability of the woman in labour in this community is limited. There is need to interact with relatives and communities of women in labour and facilitate positive companionship during labour  to improve birth outcomes [47].

### Strength and limitations

This study had some limitations. The study was conducted in one region in Uganda which may affect transferability of findings but we believe that the situation may be similar in other settings within the country and region. Women and community members were not part of the study population and therefore the community’s expectations and perspectives regarding management of foetal distress and BA should be viewed in that light.

*Strength of the study.* This study is among the few that describe health care workers’ experiences of managing foetal distress and BA at different levels of care in a low resource setting. While many studies have looked at BA management quantitatively, we believe that this study provides a deeper understanding of the comprehensive set of problems from the perspective of health care workers. Secondly, this study was conducted among different cadres of health care workers at all levels of health facilities with maternity services in the Ugandan health care system. We describe the care at each level and show the gaps and challenges of management of foetal distress and BA. Thirdly, initial data analysis was performed by two authors who are familiar with the data and setting where the study was conducted while four authors reviewed and decided on the final categories. To ensure trustworthiness, participant’s quotes have been presented together with the results.

## Conclusion and implications for practice

Management of foetal distress and BA appeared to be a big challenge to health care workers in the study setting. To deal with lack of resources, a poor referral system, and high expectations from woman and family were major challenges. We highlight some health system challenges that could be targeted for improvement of care in an effort to accelerate achievement of the *Every Newborn Action Plan’s (ENAP)* target for neonatal mortality of less than 12 deaths per 1000 live births by 2025 [2]. We recommend establishment of clear guidelines and standards for foetal distress and BA at different levels of maternity care. We believe that creating special care units for unstable/sick newborns at the lowest level of maternity care could greatly reduce the rates of undesirable outcomes. The referral system requires urgent attention from enabling efficient transportation to provision of necessary supplies and equipment for management of foetal distress and BA. Dialogue with community members and families about abuse, mistreatment and positive support and companionship during labour and birth should be implemented. Research into innovative transport mechanisms is needed to improve this aspect of the referral system.

## Data Availability

Data is available on reasonable request from the corresponding author.

## Appendix 1

See Table 2.

**Table 2:** Analysis framework for the category “understanding of and actions for foetal distress and birth asphyxia”

Open coding	Sub-categories	Category
Defining foetal distress	Knowledge of foetal distress	Managing foetal distress and birth asphyxia
Diagnosis of foetal distress		
Confirmation of foetal distress		
IV fluids/rehydration	Interventions for foetal distress	
Lateral positioning and repositioning		
Oxygen administration to mother		
Referral to a higher level facility		
Expediting delivery		
Reassuring the mother		
Consultation and team work		
Failure to initiate breathing	Knowledge of birth asphyxia	
newborn not breathing well		
failure to gain normal respirations		
newborn is blue, baby is floppy		
Apgar score less than 7		
Apgar score of 3 to 1 are difficult		
Severity of birth asphyxia		
Resuscitation	Interventions for birth asphyxia	
Suctioning of the newborn		
Using ambubag to resuscitate		
Keep the newborn warm		
Giving 10% dextrose		
Using drugs like aminophylline		
Giving oxygen		
Referral to higher facility		

## Abbreviations

BA: Birth asphyxia
ENAP: Every newborn action plan
HBB: Helping babies breathe
LMIC: Low and middle income countries
UCG: Uganda Clinical Guidelines
WHO: World Health Organisation
